# Hydrogen Sulfide Regulates SERCA2a Ubiquitylation via Muscle RING Finger-1 S-Sulfhydration to Affect Cardiac Contractility in db/db Mice

**DOI:** 10.3390/cells11213465

**Published:** 2022-11-02

**Authors:** Shuo Peng, Dechao Zhao, Qianzhu Li, Mengyi Wang, Shiwu Zhang, Kemiao Pang, Jiayi Huang, Fanghao Lu, He Chen, Weihua Zhang

**Affiliations:** 1 Department of Pathophysiology, Harbin Medical University, Harbin 150081, China; 2 Department of Cardiology, First Affiliated Hospital of Harbin Medical University, Harbin 150001, China; 3 Department of Forensic Medicine, Harbin Medical University, Harbin 150081, China; 4 Key Laboratory of Cardiovascular Medicine Research, Harbin Medical University, Ministry of Education, Harbin 150001, China

**Keywords:** hydrogen sulfide, diabetic cardiomyopathy, SERCA2a, MuRFl, S-sulfhydration

## Abstract

Hydrogen sulfide (H_2_S), as a gasotransmitter, is involved in various pathophysiological processes. Diabetic cardiomyopathy (DCM) is a major complication of diabetes mellitus (DM), which leads to structural and functional abnormalities of the myocardium and eventually causes heart failure (HF). Systolic and diastolic dysfunction are fundamental features of heart failure. SERCA2a, as a key enzyme for calcium transport in the endoplasmic reticulum (ER), affects the process of myocardial relaxation and contraction. H_2_S can protect the cardiac function against diabetic hearts, however, its mechanisms are unclear. This study found that exogenous H_2_S affects cellular calcium transport by regulating the H_2_S/MuRF1/SERCA2a/cardiac contractile pathway. Our results showed that, compared with the db/db mice, exogenous H_2_S restored the protein expression levels of CSE and SERCA2a, and the activity of SERCA2a, while reducing cytosolic calcium concentrations and MuRF1 expression. We demonstrated that MuRF1 could interact with SERCA2a via co-immunoprecipitation. Using LC-MS/MS protein ubiquitylation analysis, we identified 147 proteins with increased ubiquitination levels, including SERCA2a, in the cardiac tissues of the db/db mice compared with NaHS-treated db/db mice. Our studies further revealed that NaHS administration modified MuRF1 S-sulfhydration and enhanced the activity and expression of SERCA2a. Under hyperglycemia and hyperlipidemia, overexpression of the MuRF1-Cys44 mutant plasmid reduced the S-sulfhydration level of MuRF1 and decreased the ubiquitination level of SERCA2a and the intracellular Ca^2+^ concentration. These findings suggested that H_2_S modulates SERCA2a ubiquitination through MuRF1 S-sulfhydration of Cys44 to prevent decreased myocardial contractility due to increased cytosolic calcium.

## 1. Introduction

The number of patients with diabetes mellitus, who are predisposed to cardiovascular diseases, such as atherosclerotic vascular diseases and diabetic cardiomyopathy, has been increasing worldwide in the past three decades [1,2]. Diabetic cardiomyopathy is a DM-induced pathophysiological condition resulting in structural and functional abnormalities of the myocardium [3]. According to a population-based study, the risk of heart failure is increased two- to three-fold in patients with DM [4]. At an early stage, patients with DM can present with normal systolic contribution, but impaired diastolic function, which is termed HF with a preserved ejection fraction (HFpEF); however, at a late stage, these patients have a decrease in right and left ventricular ejection fractions.

Cardiac contractility is regulated through the precise interplay between several cellular calcium transport protein complexes, including the L-type Ca^2+^ channel, ryanodine receptor, sarcoplasmic reticulum Ca^2+^-ATPase (SERCA2a), and sodium-calcium exchanger, among others [5]. SERCA2a plays a major role in cardiac excitation-contraction coupling and cardiac contractility. Increasing evidence has demonstrated that SERCA2a expression and activity are decreased in different pathophysiological conditions, including diabetes [6]. However, the mechanism of the decrease in SERCA2a expression and activity is still unclear. Many studies have reported that SERCA2a is modulated by several posttranslational modifications (PTMs), including SUMOylation, phosphorylation, acetylation, ubiquitination, and nitration [7,8].

Hydrogen sulfide (H_2_S) is considered a by-product of metabolic processes in mammalian cells. Three enzymes generate H_2_S from L-cysteine, via independent reactions: cystathionine γ lyase (CSE), cystathionine-β-synthase (CBS), and 3-mercaptopyruvate sulfurtransferase (3-MST) [9]. Many studies have reported that all three enzymes are ubiquitous in tissues and that H_2_S impacts almost all cellular processes, including the regulation of vascular tone and blood pressure, anti-inflammation, and antioxidation [10,11]. Our previous studies have demonstrated that exogenous H_2_S can ameliorate cardiac contractility in db/db mice [12,13]; however, the mechanism is unclear. In this study, we examined the effects of H_2_S on SERCA2a ubiquitination in db/db mice to restore its expression and activity. We demonstrate that inhibiting the ubiquitination level of SERCA2a is a new therapeutic target for H_2_S in the treatment of heart failure.

## 2. Materials and Methods

### 2.1. Animal Model and Treatment Protocols

Homozygous male and female 8-week-old db/db mice (*n* = 50) and 8-week-old C57BL/6 mice (*n* = 60) were used in this experiment, provided by the Animal Laboratory Centre of Nanjing University. They are grown in an ultra-clean environment with a 12-h light-dark cycle at 22–24 °C. The experimental animals were randomly divided into four groups. Half of the C57BL/6 mice and db/db mice were intraperitoneally injected with NaHS (80 μmol/L/kg, Sigma-Aldrich, Saint Louis, MO, USA), every two days for 18 weeks. Meanwhile, the remaining half were injected with an equal volume of normal saline. All animal experiments were performed in accordance with the Guide for the Care and Use of Laboratory Animals, published by the National Institutes of Health of China, and approved by the Animal Care Committee of Harbin Medical University.

### 2.2. Echocardiography Analysis

The left ventricular function of the mice was examined by transthoracic echocardiography using an ultrasound machine (GE vivid 7 medical). The experimental mice were anesthetized by an intraperitoneal injection of avertin (240 mg·kg^−1^, Sigma-Aldrich), and the mice were fixed in the supine position. Ejection fraction (EF%) and short-axis shortening rate (FS%) were detected by transthoracic echocardiography. Each parameter was calculated using the M-mode view.

### 2.3. LC-MS/MS Analysis

To identify the lysine ubiquitylome of the cardiac tissues of the db/db mice and the mice treated with NaHS, the above extracted proteins were digested with protease. Kub peptide enrichment and HPLC-MS/MS analysis were then performed using the methods previously described. Intensive bioinformatics analysis was then carried out to annotate those quantifiable lysine ubiquitinated targets, including protein annotation, functional classification, functional enrichment, functional enrichment-based cluster analysis, etc.

### 2.4. Glucose Tolerance Test Analysis

The experimental mice were intraperitoneally injected with D-glucose at 2 g/kg body weight. Subsequently, tail vein blood was collected at the corresponding time points, and blood glucose was determined using a glucometer.

### 2.5. Immunoprecipitation

Agarose beads (Protein G Plus Agarose, Santa Cruz, CA, USA) were added to the samples at 4 °C for 30 min, take the supernatant after centrifugation. Agarose beads and antibodies (anti-SERCA2a or anti-MuRF1) were added in proportion, at 4 °C overnight with gentle rotation. After centrifugation, sepharose beads were resuspended in a buffer containing PMSF and washed three times. The precipitation was diluted with loading buffer. Protein-protein interactions of samples were detected by Western blot.

### 2.6. Immunoblot Analysis

Western blotting was performed as described previously. Primary antibodies included anti-CSE (Proteintech, Rosemont, IL, USA), anti-SERCA2a (Invitrogen, Carlsbad, CA, USA), anti-Ubiquitin (Proteintech), anti-eIF2α (Proteintech), anti-P-eIF2α (Ser51, Cell Signaling Technology, Danvers, MA, USA), anti-PERK (Proteintech), anti-P-PERK (Thr-980, Cell Signaling Technology), anti-Bip (Proteintech), anti-CHOP (Proteintech), anti-MuRF1 (Proteintech), anti-Caspase3 (Proteintech), anti-β-Tubulin (Proteintech). The primary antibody was incubated overnight at 4 °C. Density measurements were performed using the image processing and analysis program alphaview SA (3.3.0, provided by ProteinSimple), and data were expressed in relative units.

### 2.7. Measurement of SERCA2a Activity

SERCA2a activity was detected using the ultra-micro Ca^2+^-ATPase kit (Nanjing Jiancheng Bioengineering Institute, Nanjing, China). ATPase can decompose ATP to generate ADP and inorganic phosphate (Pi), and the amount of Pi can be used to determine the level of ATPase activity. The extracted protein samples were equally divided into two parts, the samples in the absence (total activity) or the presence of 5 μM thapsigargin (activity of thapsigargin-insensitive calcium pumps, Sigma-Aldrich). Then follow the steps required by the kit. The thapsigargin-sensitive Ca^2+^-ATPase (SERCA2a-ATPase) was calculated by subtraction of thapsigargin-insensitive Ca^2+^-ATPase activity from total activity. 

### 2.8. S-Sulfhydration Assay

The cardiac tissue or cardiomyocytes were homogenized in HEN buffer [250 mM HEPES-NaOH (pH 7.7), 1 mM EDTA, and 0.1 mM neocuproine] and 100 μM deferoxamine, centrifuged at 13,000× *g* for 30 min at 4 °C. Cell lysates were incubated with blocking buffer [HEN buffer adjusted to 2.5% SDS and 20 mM methyl methanethiosulfonate (MMTS)] for 20 min at 50 °C with frequent vortexing. Then, we added acetone and precipitated at −20 °C for 30 min. After the removal of acetone, the sample was resuspended in a blocking buffer containing 1% SDS, followed by the addition of 4 mM biotin-HPDP. We added streptavidin-agarose beads and incubated them for 3h at 25 °C. Then, the samples were washed with HENS buffer. Biotinylated proteins were eluted by SDS-PAGE and subjected to Western blot analysis using an anti-MuRF1 antibody (Proteintech). HEPES, NaOH, EDTA, neocuproine, deferoxamine, SDS, MMTS, biotin-HPDP, and streptavidin-agarose beads were purchased from Sigma-Aldrich. 

### 2.9. Neonatal Rat Cardiomyocytes Culture

Cultures of neonatal rat cardiomyocytes were prepared according to previously described methods. The hearts were prepared from 2- to 3-day-old neonatal Sprague-Dawley rats (Animal Research Institute of Harbin Medical University, China), and the hearts were cut into pieces of less than 1 mm^3^ in 0.25% trypsin. The hearts pieces were incubated with trypsin at 37 °C for 8 min, terminating the digestion with an equal volume of Dulbecco’s modified Eagle’s medium [DMEM, containing 10% (*v*/*v*) fetal bovine serum (HyClone, Logan, UT, USA)], the digestion was repeated 6 times. Cells were collected by centrifugation at 2000× *g* for 10 min, then resuspended in DMEM and incubated at 37 °C in humidified air containing 5% CO_2_ for 1h. Attached cells were discarded and unattached cells were cultured in new medium. Change the culture medium every 2 days.

### 2.10. Cell Experiment

The cultured neonatal rat cardiomyocytes were divided into the following groups and treatments: control group (low glucose, LG, 5.5 mM), high glucose (HG, 40 mM) + Oleate (Ole, 200 µM) + Palmitate (Pal, 200 µM), HG + Ole + Pal + NaHS (H_2_S donor, 100 μM), HG + Ole + Pal + propargylglycine (PPG, a CSE inhibitor, 10 nM), HG + Ole + Pal + MG132 (20 μM, an inhibitor of proteasome), HG + Ole + Pal + 4-PBA (5 mM, an inhibitor of endoplasmic reticulum stress); control + DTT (1 mM, an inhibitor of disulfide bond), HG + Ole + Pal + NaHS + DTT. NaHS, palmitate, oleate, PPG, DTT, MG132, and 4-PBA were purchased from Sigma-Aldrich. 

### 2.11. Point Mutation

Adenoviruses expressing GFP, SERCA2a-GFP, and MuRF1-GFP were purchased from Cyagen Biosciences Inc. (Santa Clara, CA, USA). The 460 Lysine site of SERCA2a (ATP2A2) was mutated to alanine and the 44 cysteine site of MuRF1 was mutated to alanine. This was followed by the insertion of full-length rat SERCA2a with a single mutation of lysine 460 to alanine and GFP cDNA or full-length rat MuRF1 of cysteine 44 to alanine and GFP cDNA were inserted into pM vector (Cyagen Biosciences) between the Kozak and T2A sites. The adenovirus was added directly to NRCMs, and the new fresh medium was added after 4–6 h for transfection. The NRCMs were treated in different conditions 24 h after transfection, and the related proteins were detected by Western blotting.

### 2.12. H_2_S Level Detection with 7-Azido-4-Methylcoumarin

The fluorescence response of H_2_S in cardiac tissue and neonatal rat cardiomyocytes (NRCMs) was detected using the H_2_S-specific detection probe C-7Az (7-azido-4-methylcoumarin, Sigma-Aldrich). Frozen sections of db/db cardiac tissues or NRCMs were incubated with H_2_S-specific detection probe C-7Az (50 μmol/L) in phosphate-buffered saline (PBS) for 30 min at 37 °C. These were washed three times with PBS. Fluorescence microscopy (Olympus, XSZ-D2, Tokyo, Japan) was used to detect the fluorescence responses of H_2_S in the samples.

### 2.13. Measurement of Intracellular Levels of Polysulfide

Using the polysulfide-specific fluorescent probe SSP4 (Dojindo, Kumamoto, Kyushu, Japan), the neonatal rat cardiomyocytes were detected for polysulfides, SSP4 (50 μmol/L) probe in serum-free DMEM medium at 37 °C for 30 min. These were washed 3 times with PBS. Fluorescence microscopy detects fluorescence levels. Fluorescence microscopy (Olympus, XSZ-D2) was used to detect the fluorescence responses of polysulfide in the samples.

### 2.14. [Ca^2+^]_i_ Assay

The intracellular Ca^2+^ concentration was detected using the Fura 2-AM fluorescent probe (Invitrogen). The neonatal rat cardiomyocytes (NRCMs) were plated into black 96-well plates. After being cultured for 48 h, the medium was changed into 100 μL fresh DMEM medium containing 10% (*v*/*v*) fetal bovine serum, and the cells were subjected to corresponding experimental treatment according to the experimental requirements and then incubated for 48 h. Subsequently, the NRCMs were washed 3 times with PBS and incubated with a 2 μM Fura-2-AM fluorescent probe for 30 min at 37 °C. After incubation, the cells were washed 3 times with PBS and the fluorescence intensity was measured by a Fluorescence Spectrophotometer (Cary Eclipse, Palo Alto, CA, USA) at an emission wavelength of 510 nm and excitation wavelengths of 340 nm and 380 nm.

### 2.15. Caspase3 Activity Assays

Caspase3 activity in neonatal rat cardiomyocytes (NRCMs) was detected using the Caspase3 Activity Assay Kit (Beyotime, Shanghai, China). The NRCMs were lysed using lysis buffer, the homogenized supernatant was collected by centrifuging at 13,500× *g* for 30 min, followed by measuring protein concentration using the BCA protein assay kit, and cell lysates were incubated with Ac-DEVD-pNA (2 mM) was incubated at 37 °C for 2 h. After incubation, the absorbance was read at 405 nm using a microplate reader (BioTek, Winooski, VT, USA).

### 2.16. Statistical Analysis

The results were analyzed using Prism software package (GraphPad Software, 8.0.2, San Diego, CA, USA), and experimental data were presented as mean ± standard error (SEM). More than two groups were compared using a one-way ANOVA and Bonferroni’s correction. Differences between individual groups were analyzed using Student’s t-test. The threshold of *p* < 0.05 was designated statistically significant for all tests. 

## 3. Results

### 3.1. Alterations in Body Weight and Glucose Levels in db/db Mice

In our study, db/db mice were chosen as type two diabetes mellitus (T2DM) animal models. At the age of 26 weeks, the db/db mice were significantly overweight compared to WT control mice. Glucose concentrations and glucose tolerance were elevated in db/db mice (27.48 ± 4.84 mM). However, a significant increase in heart weight normalized to body weight was identified in diabetic mice compared to WT control mice and NaHS-treated db/db mice. These data demonstrated that db/db mice were typical T2DM animal models (Figure S1).

### 3.2. Decreased CSE Expression and the Effects of H_2_S on Cardiac Function and Structure in Mice with T2DM

**Figure 1:**
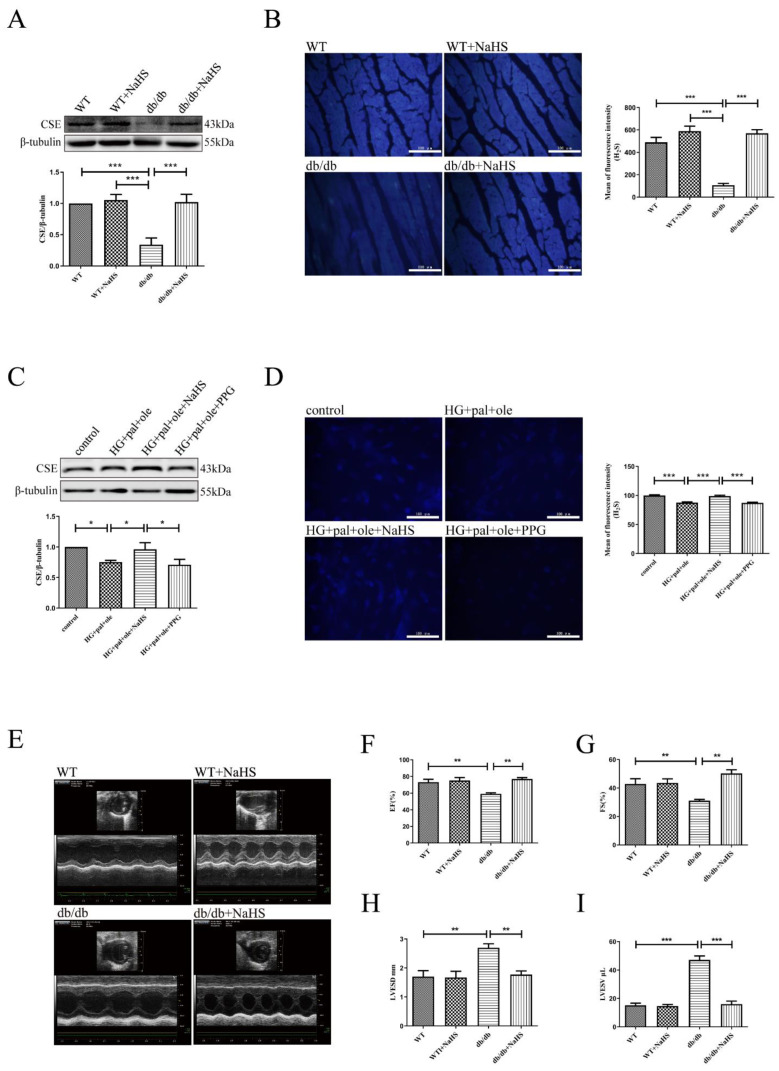
Exogenous H_2_S ameliorated cardiac function in db/db mice. (**A**) CSE expression was detected by Western blot analysis in the cardiac tissues (*n* = 5). Scale bar: 100 μm. (**B**) In cardiac tissues of db/db mice, H_2_S content was detected by 7-azido-4-methylcoumarin (*n* = 5). (**C**) CSE expression was detected by Western blot analysis in the NRCMs (*n* = 5). (**D**) In NRCMs, H_2_S content was detected by 7-azido-4-methylcoumarin (*n* = 6). Scale bar: 100 μm. (**E**) Representative M-mode echocardiographic of mice hearts. (**F**) Quantitative analysis of Ejection fraction by echocardiography (*n* = 4). (**G**) Quantitative analysis of Fraction shortening by echocardiography (*n* = 4). (**H**) Quantitative analysis of LV end-systolic dimension by echocardiography (*n* = 4). (**I**) Quantita-tive analysis of LV end-systolic volume by echocardiography (*n* = 4). All values are presented as means ± standard errors, * *p* < 0.05, ** *p* < 0.01, *** *p* < 0.001.

At the age of eight weeks, the db/db mice were treated with NaHS (80 μmol/kg) for 18 weeks. Our previous study demonstrated that the expression of CSE (a key enzyme of H_2_S generation) in cardiac tissues was significantly decreased in STZ-induced T1DM [14]. In this study, we examined CSE expression and H_2_S contents in cardiac tissues in T2DM. Our results showed that the expression of CSE and the contents of H_2_S, which was detected by 7-azido-4-methylcoumarin (a specific fluorescent probe for H_2_S), were significantly decreased in T2DM mice compared to WT control mice and the db/db mice treated with NaHS (Figure 1A,B). In addition, the NRCMs were treated with 40 mM glucose, 200 μM palmitate, and 200 μM oleate as a cellular model to mimic T2DM. Similar patterns of CSE expression alteration and H_2_S contents were consistently observed in treated NRCMs and T2DM mice (Figure 1C,D).

Echocardiography was used to determine the effects of exogenous H_2_S on cardiac functions. As illustrated in Figure 1E–I, the ejection fraction (EF) and fractional shortening (FS) were substantially lower in the db/db mice than in the control and db/db mice treated with exogenous H_2_S. Meanwhile, LVESD and LVESV were significantly increased in the db/db mice compared with the control and db/db mice. Taken together, exogenous H_2_S significantly improved cardiac function in T2DM mice.

### 3.3. Exogenous H_2_S Restored SERCA2a Protein Levels and Activity in db/db Mice

**Figure 2:**
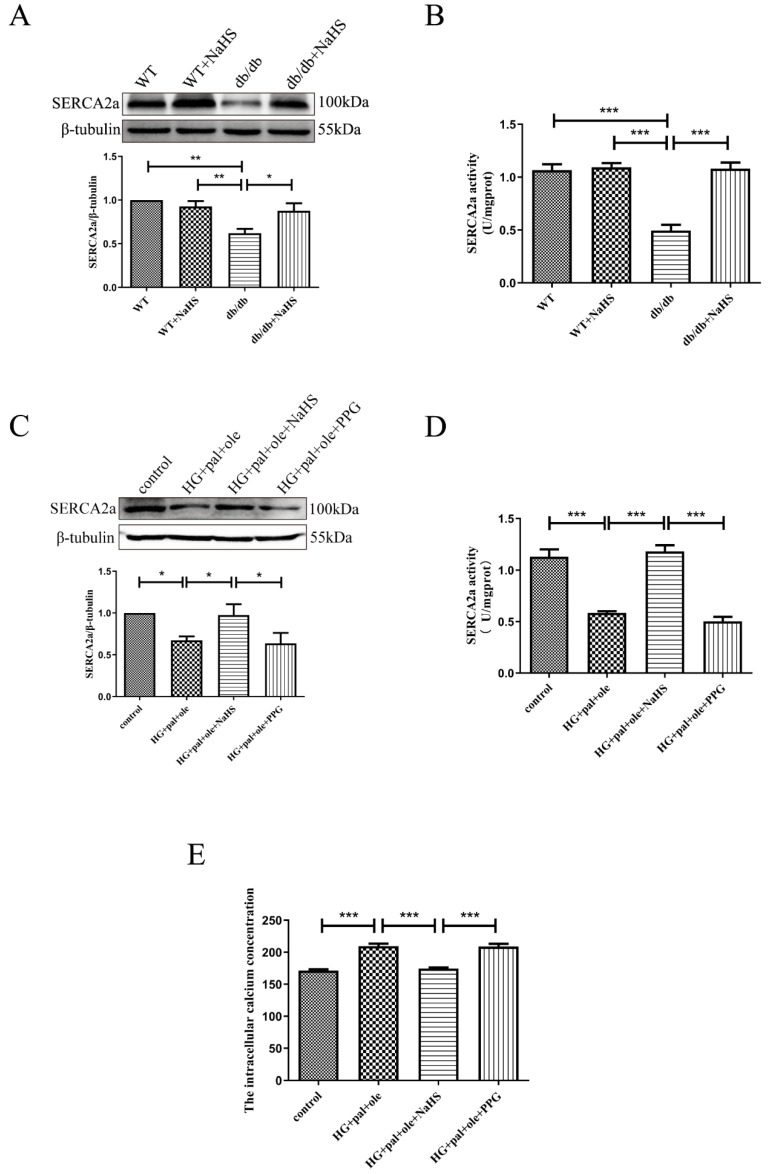
Exogenous H_2_S improved SERCA2a protein level and activity in db/db mice. (**A**) SERCA2a expression was detected by Western blot analysis in the cardiac tissues (*n* = 6). (**B**) The results of SERCA2a activity in mice hearts (*n* = 5). (**C**) SERCA2a expression was detected by Western blot analysis in NRCMs (*n* = 5). (**D**) The results of SERCA2a activity in NRCMs (*n* = 4). (**E**) Intracellular calcium concentration ([Ca^2+^]_i_) was detected by Fura-2 in NRCMs (*n* = 6). All values are presented as means ± standard errors, * *p* < 0.05, ** *p* < 0.01, *** *p* < 0.001.

We explored whether H_2_S could ameliorate cardiac function by investigating SERCA2a expression and activity. SERCA2a, which is the primary cardiac isoform and is involved in the sequestration of Ca^2+^ into the sarcoplasmic reticulum (SR) during diastole, has been reported to play a predominant role in regulating calcium homeostasis in cardiomyocytes [15]. As shown in Figure 2A,B, the cardiac protein level and activity of SERCA2a were predominantly decreased in db/db mice, relative to the control mice and db/db mice treated with exogenous H_2_S. Similarly, SERCA2a protein levels and activity were also significantly reduced in the NRCMs treated with glucose, palmitate, and oleate compared to cells in the control and NaHS groups, and PPG (propargylglycine), an inhibitor of CSE, also decreased the expression of SERCA2a (Figure 2C,D). Meanwhile, the intracellular calcium concentration ([Ca^2+^]_i_) was also measured in the NRCMs loaded with Fura-2 (Figure 2E). HG + pal + ole and PPG treatment caused Ca^2+^ overload compared to control and exogenous H_2_S treatments. The above results revealed that H_2_S improved the protein level and activity of SERCA2a in db/db mice.

### 3.4. Identification of SERCA2a Ubiquitylation by LC–MS/MS Analysis

**Figure 3:**
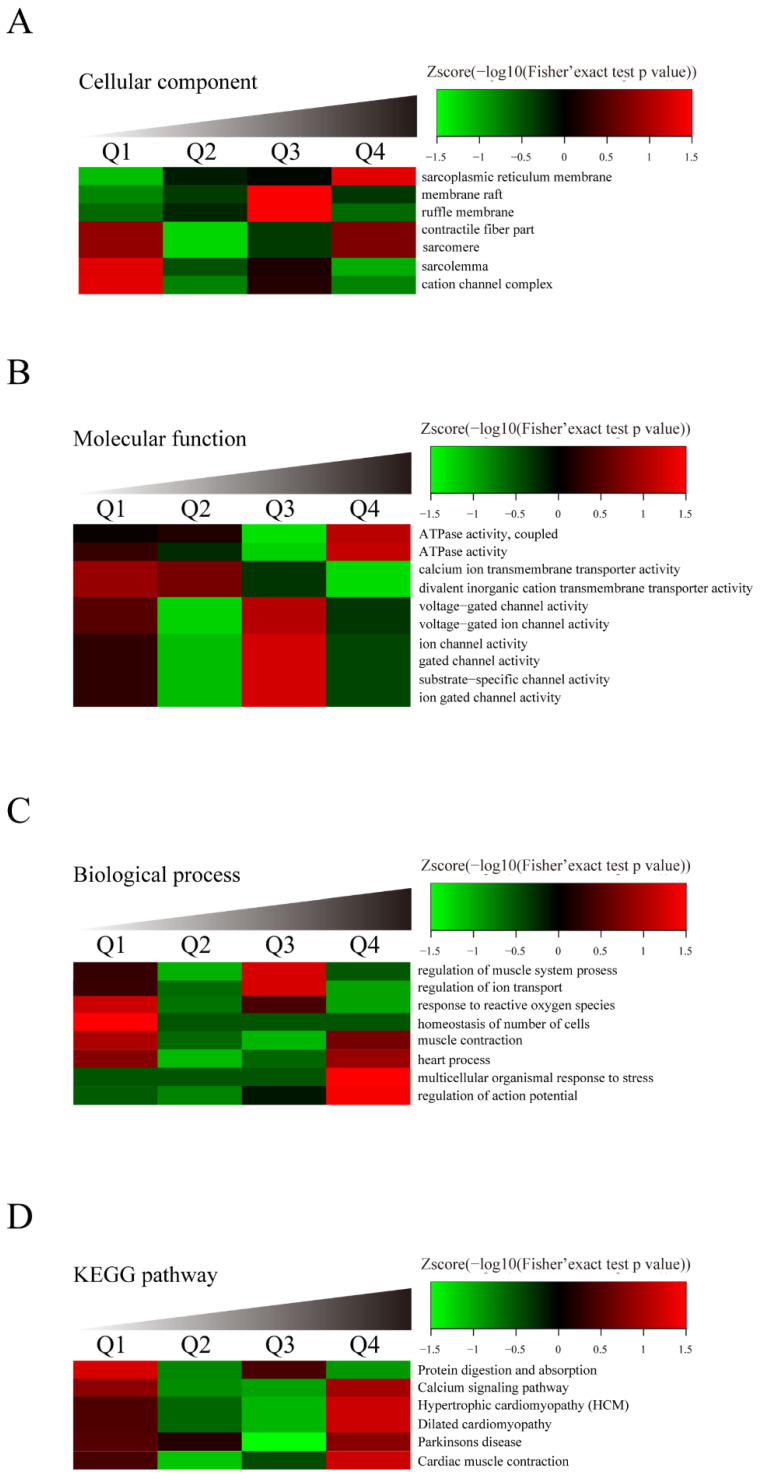

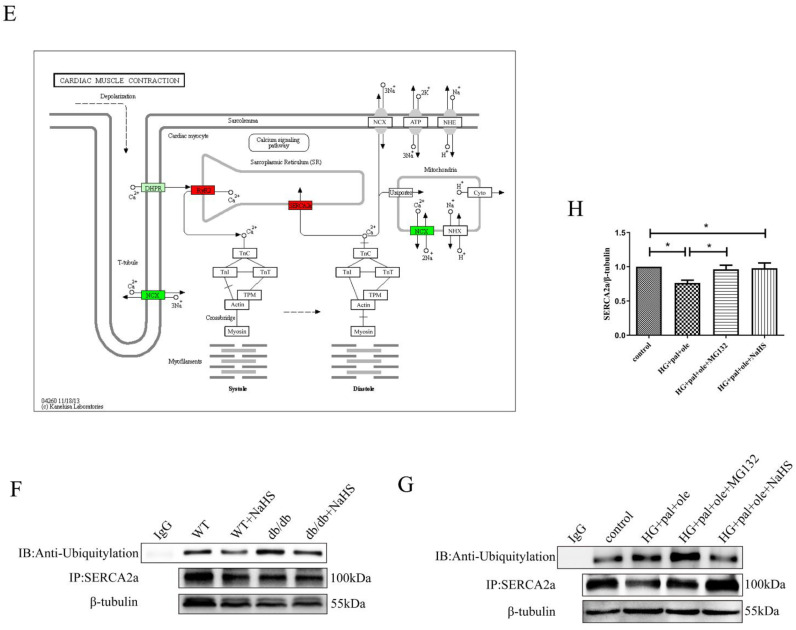
Exogenous H_2_S upregulated SERCA2a in cardiac tissues of db/db mice via inhibiting ubiquitin-proteasome pathway. Affinity enrichment was normalized using KEGG standard peptides, followed by a label-free quantitative proteomic strategy for quantitative lysine ubiquitination analysis of mouse cardiac tissue. GO annotation divides proteins into three categories: cell compartment, molecular function, and biological process. Kub protein quantized in this study is divided into four quantiles according to the quantized proportion: Q1 (0~15%), Q2 (15~50%), Q3 (50~85%), and Q4 (85~100%). Calculate the relative fold change between the db/db model mice and the db/db+NaHS mice. Green means score < 0, and the ratio is lower than the average. Red indicates that the score > 0, and the ratio is greater than the average value. (**A**) Cellular Component. (**B**) Molecular Function. (**C**) Biological Process. (**D**) KEGG pathway analysis was identified by bioinformatic analysis. (**E**) The pathway obtained from KEGG pathway enrichment analysis. The proteins in bright green are down-regulated and the proteins in red are up-regulated. (**F**) Cardiac tissue lysate was immunoprecipitated with anti-SERCA2a antibody and then immunoblotted with antibodies specific for Ubiquitylation. (**G**) The NRCMs were immunoprecipitated with an anti-SERCA2a antibody and then immunoblotted with antibodies specific for Ubiquitylation. (**H**) After being treated with MG132, the expression of SERCA2a was assessed by Western blotting in the NRCMs. Values are presented as the mean ±standard errors from *n* = 6 replicates. * *p* < 0.05.

With the above results indicating the significant role of H_2_S in regulating SERCA2a protein levels, we explored how H_2_S can restore the SERCA2a protein level. To gain insight into this issue, we performed an LC–MS/MS proteomic analysis to demonstrate lysine ubiquitylation in the cardiac tissues of the db/db mice and the db/db mice treated with NaHS. More than 147 identified proteins showed lysine ubiquitylation, 62 of which were hyperubiquitylated in the hearts of the db/db mice compared with the hearts of the NaHS-treated db/db mice. The results of molecular function biological process and KEGG pathway-based cluster analyses [16,17] are shown in Figure 3A–D. Proteins modified by ubiquitylation were found to be involved in regulating heart contraction and calcium signaling. ATPase activity was upregulated in the cardiac tissues of the db/db mice compared with the NaHS-treated db/db mice. The LC–MS/MS results showed that the ubiquitylation level of SERCA2a in the db/db mice increased 5.6-fold compared to that in the NaHS-treated db/db mice. As illustrated in Figure 3E, an altered SERCA2a protein level was a key element in regulating cardiac function in db/db mice.

To further confirm the LC–MS/MS results, SERCA2a ubiquitination was tested by immunoprecipitation. Our data showed that the levels of ubiquitinated SERCA2a in the hearts of the db/db mice were higher than those in the NaHS-treated db/db mice (Figure 3F). Similarly, SERCA2a ubiquitylation was upregulated in the NRCMs treated with HG + pal + ole, compared to the NRCMs treated with NaHS. MG132 (10 μM), an inhibitor of the proteasome, was added to increase the ubiquitylation level of SERCA2a (Figure 3H).

**Figure 4:**
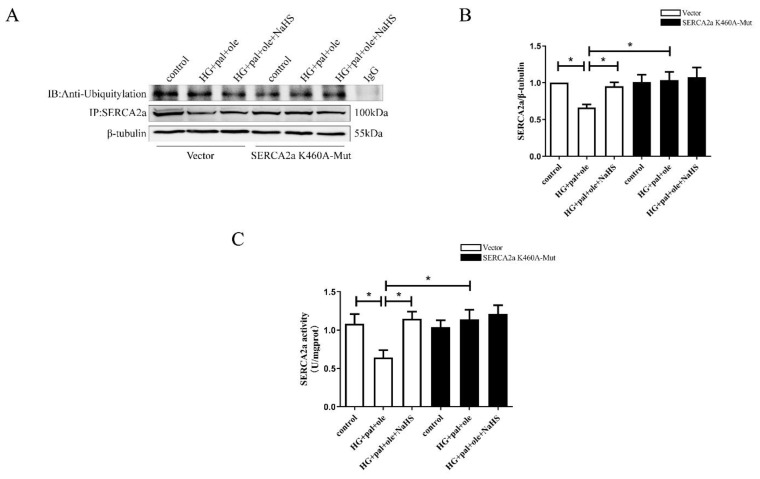
Exogenous H_2_S downregulated SERCA2a ubiquitylation at Lys460. After transfection with plasmids containing wild-type SERCA2a or SERCA2a mutated at Lys460 for 24 h, the NRCMs were treated with high glucose, palmitate, and oleate for 48 h in the presence or absence of NaHS. (**A**) The NRCMs were immunoprecipitated with an anti-SERCA2a antibody and then immunoblotted with antibodies specific for Ubiquitylation after transfection. (**B**) The expression of SERCA2a was assessed by Western blotting in the NRCMs after transfection (*n* = 7). (**C**) The results of SERCA2a activity in the NRCMs after transfection (*n* = 6). All values are presented as means ± standard errors, * *p* < 0.05.

SERCA2a is responsible for the reuptake of cytosolic Ca^2+^ into the sarcoplasmic reticulum (SR) of cardiomyocytes, which enables cardiac muscle relaxation and determines how much Ca^2+^ can be released for contraction to further modulate cardiac contractility [8,18]. Growing evidence demonstrates that SERCA2a isoforms are regulated by various posttranslational modifications (PTMs), including SUMOylation, phosphorylation, acetylation, ubiquitination, and nitration [19]. Ubiquitination sites (Lys 169, Lys 460, Lys 476, Lys 514, Lys 533, Lys 541, Lys 611, Lys 628, Lys 650, Lys 757, and Lys 995) were identified in SERCA2a [7]. Three cytoplasmic domains include the phosphorylation domain (PD), the nucleotide domain (ND), and the actuator domain (AD), which regulate ATP binding and autophosphorylation [20]. Lysine 460 is located in the nucleotide domain of SERCA2a. We constructed a SERCA2a mutation plasmid in which lysine 460 was mutated to alanine. Next, we transfected the NRCMs with wild-type SERCA2a or SERCA2a-K460A overexpression plasmids. After transfection, no difference in the ubiquitylation level of SERCA2a or the activity of SERCA2a was observed in the HG + pal + ole group compared to the NaHS group (Figure 4A–C).

### 3.5. MuRF1 Is Required for SERCA2a Degradation

**Figure 5:**
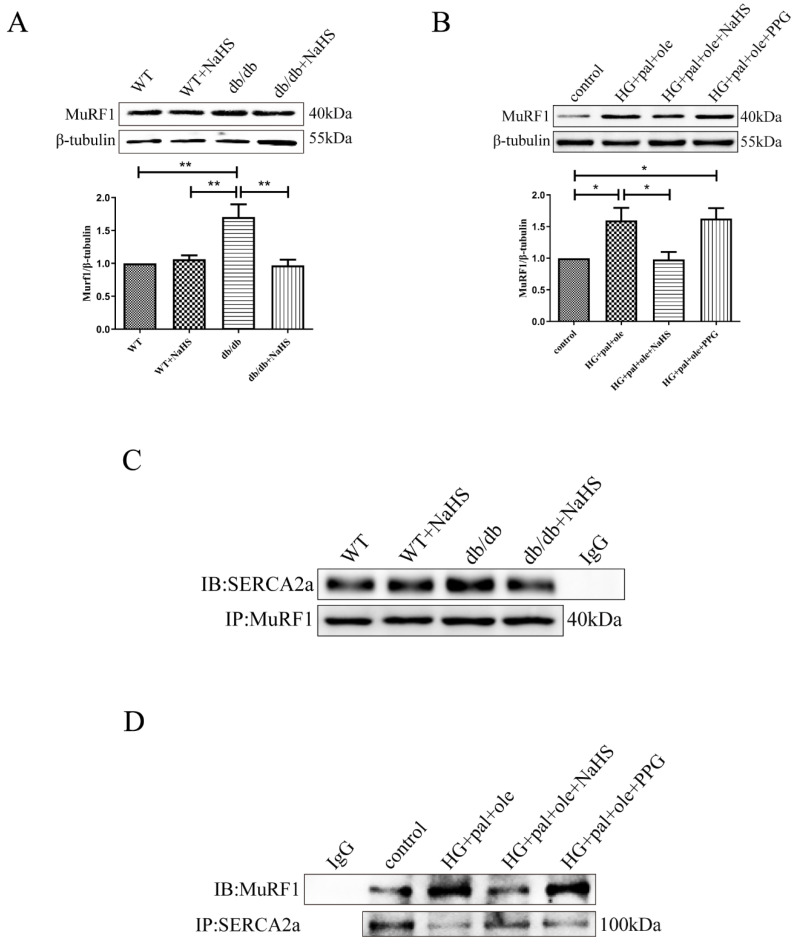
Exogenous H_2_S downregulates the interaction of SERCA2a with MuRF1. (**A**) SERCA2a expression was detected by Western blot analysis in the cardiac tissues (*n* = 4). (**B**) SERCA2a expression was detected by Western blot analysis in the NRCMs (*n* = 3). (**C**) Cardiac tissue lysate was immunoprecipitated with anti MuRF1 antibody and then immunoblotted with antibodies specific for SERCA2a. (**D**) The NRCMs were immunoprecipitated with an anti-SERCA2a antibody and then immunoblotted with antibodies specific for MuRF1. All values are presented as means ± standard errors, * *p* < 0.05, ** *p* < 0.01.

MuRF1 was identified as a muscle-specific RING finger protein that is bound to the kinase of sarcomeric proteins [21]. MuRF1, as an E3 ligase, was highly expressed in skeletal muscle and cardiac muscle. Several studies have reported that increases in proteolysis occur in various cardiac disorders, including those attributable to sepsis, diabetes, and starvation [22,23,24]. In this study, MuRF1 expression was upregulated in the db/db mice compared with the NaHS-treated db/db mice and control groups. A similar alteration occurred in the cellular models (Figure 5A,B). Considering the relationship between MuRF1 and SERCA2a, we hypothesized that MuRF1 was involved in SERCA2a ubiquitination. Co-IP results confirmed the interaction between MuRF1 and SERCA2a in animal and cellular models (Figure 5C,D).

### 3.6. H_2_S Promoted MuRF1 S-Sulfhydration to Affect Its Activity

**Figure 6:**
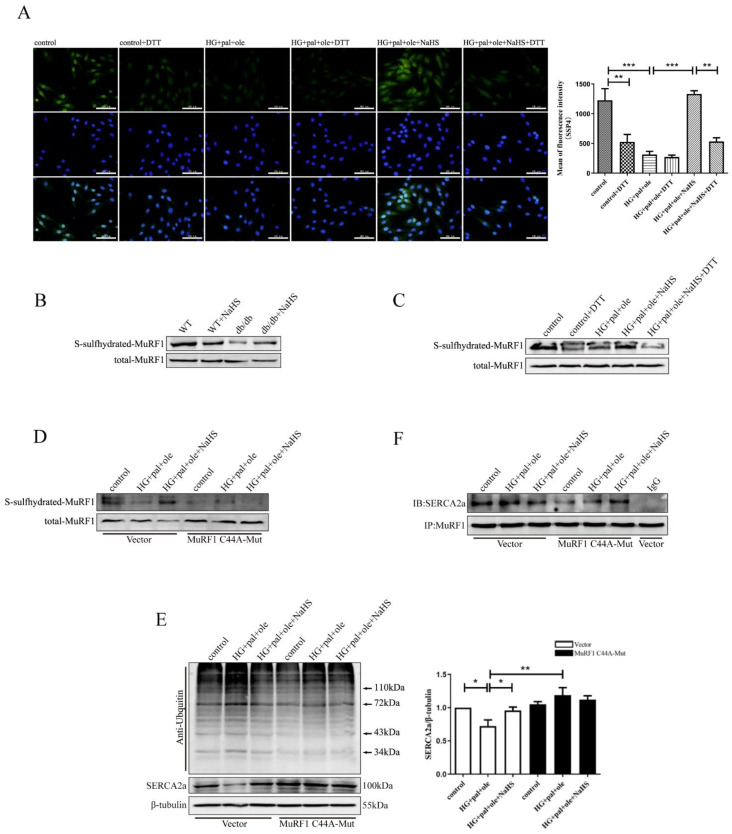
MuRF1 Cys44 site mutation increases SERCA2a protein content. (**A**) Detection of intracellular polysulfide levels in the NRCMs using fluorescent probe SSP4 (*n* = 3). Scale bar: 100 μm. (**B**) The level of S-sulfhydration of MuRF1 in cardiac tissues was detected using the biotin switch method. (**C**) The level of S-sulfhydration of MuRF1 in the NRCMs was detected using the biotin switch method. After transfection with plasmids containing wild-type MuRF1 or MuRF1 mutated at Cys44 for 24 h, the NRCMs were treated with high glucose, palmitate, and oleate for 48 h in the presence or absence of NaHS. (**D**) The level of S-sulfhydration of MuRF1 in NRCMs after transfection was detected using the biotin switch method. (**E**) The expression of Ubiquitin and SERCA2a (*n* = 5) was assessed by Western blotting in the NRCMs after transfection. (**F**) The NRCMs after transfection were immunoprecipitated with anti-MuRF1 antibody and then immunoblotted with antibodies specific for SERCA2a. All values are presented as means ± standard errors, * *p* < 0.05, ** *p* < 0.01, *** *p* < 0.001.

The data presented above suggested that MuRF1 upregulation in the db/db mice produced deleterious effects on their hearts; however, exogenous H_2_S could restore MuRF1 expression and decrease the ubiquitylation level of SERCA2a. We questioned how exogenous H_2_S modulates MuRF1 activity. Sulfhydration is a posttranslational modification on cysteine residues, where the -SH group is converted to a persulfide or -SSH group. H_2_S, as a gasotransmitter, is involved in sulfhydration modification of target proteins [25,26]. We used the fluorescent probe SSP4 to measure intracellular protein polysulfide levels. DTT (1 mmol/L), a reducing agent that reverses thiol from sulfhydration, was used to treat the NRCMs for 30 min. As shown in Figure 6A, exogenous H_2_S enhanced SSP4 fluorescence intensity compared to treatment with HG + pal + ole and DTT in the cellular models, revealing that H_2_S promotes polysulfide accumulation. To further demonstrate the effect of H_2_S on MuRF1 by S-sulfhydration, we measured the S-sulfhydration of the MuRF1 protein by a biotin switch assay. As illustrated in Figure 6B,C, the S-sulfhydration level of MuRF1 was decreased in the db/db mice and the NRCMs treated with HG + pal + ole, whereas exogenous H_2_S enhanced MuRF1 S-sulfhydration levels in animal and cellular models.

Our previous study confirmed that Cys44 is located in the RING-type zinc finger domain [27]. Cys44 plays a key role in MuRF1 activity. We mutated Cys44 to alanine, and mutants of MuRF1 Cys44 were transfected into NRCMs. We found that exogenous H_2_S could not enhance the MuRF1 S-sulfhydration level (Figure 6D). No difference in the ubiquitylation level of SERCA2a or SERCA2a expression was noted after transfecting mutants of MuRF1-Cys44 (Figure 6E,F). Taken together, our results demonstrated that H_2_S restores SERCA2a protein levels by regulating MuRF1 S-sulfhydration.

### 3.7. Exogenous H_2_S Regulates ER Stress and Apoptosis via SERCA2a Ubiquitylation

**Figure 7:**
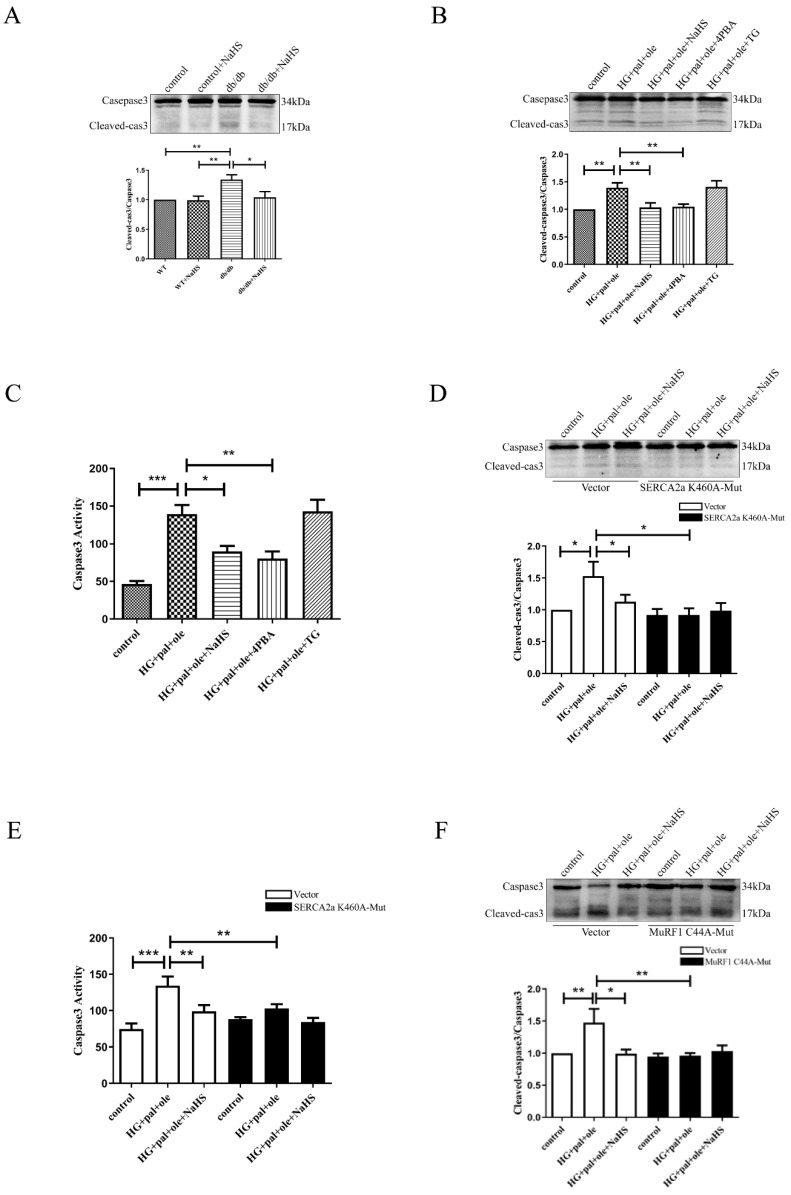
Mutation of SERCA2a Lys460 site reduces the level of apoptosis. (**A**) The expression of apoptotic marker cleave-caspase3 in cardiac tissues was tested by Western blotting (*n* = 7). (**B**) The expression of cleave-caspase3 in the NRCMs by Western blotting (*n* = 6). (**C**) The activity of Caspase3 in the NRCMs (*n* = 4). The NRCMs were transfected with plasmids containing wild-type SERCA2a or SERCA2a mutated at Lys460. (**D**) The expression of cleave-caspase3 in the NRCMs by Western blotting after transfection (*n* = 7). (**E**) The activity of Caspase3 in the NRCMs after transfection (*n* = 6). Transfection with plasmids containing wild-type MuRF1 or MuRF1 mutated at Cys44. (**F**) The expression of cleave-caspase3 in the NRCMs by Western blotting after transfection (*n* = 7). All values are presented as means ± standard errors, * *p* < 0.05, ** *p* < 0.01, *** *p* < 0.001.

The sarcoplasmic reticulum (SR) plays a crucial role in Ca^2+^ handling, and SR dysfunction results in contractile dysfunction in the heart and leads to ER stress by impairing Ca^2+^ homeostasis [28,29]. As shown in Figures S2A,B, myocardial levels of Bip, a marker of ER stress, were significantly higher in the db/db mice and the NRCMs treated with high glucose, palmitate, and oleate than in control and exogenous H_2_S groups. The levels of phospho-eIF2α, phospho-PERK, and CHOP were also significantly increased in the cardiac tissues of the db/db mice and the NRCMs in the HG + pal + ole groups compared to the control and NaHS-treated groups. 4-PBA inhibited the protein levels of BIP, phospho-eIF2α, phospho-PERK, and CHOP. Elevated intracellular Ca^2+^ provides a stimulus for the induction of ER stress, as well as cell death since a variety of kinases and signaling cascades are directly activated by Ca^2+^. We hypothesized that the decreased SERCA2a protein level was involved in cardiomyocyte apoptosis in db/db mice. Our results demonstrated that caspase-3 was obviously activated in the db/db mice and the NRCMs treated with high glucose, palmitate, and oleate compared to corresponding findings in the control and NaHS-treated groups (Figure 7A–C). After transfecting the SERCA2a-K460A plasmid, no difference in caspase-3 activation or caspase-3 activity was found (Figure 7D,E). Next, we transfected the NRCMs with wild-type MuRF1 and MuRF1-C44A. After transfection, high glucose, palmitate, and oleate did not increase the cleavage of caspase-3 compared to the control and exogenous H_2_S treatments. Taken together, our results demonstrated that H_2_S could reduce the ubiquitylation level of SERCA2a to inhibit cardiomyocyte apoptosis in db/db mice.

## 4. Discussion

**Figure 8:**
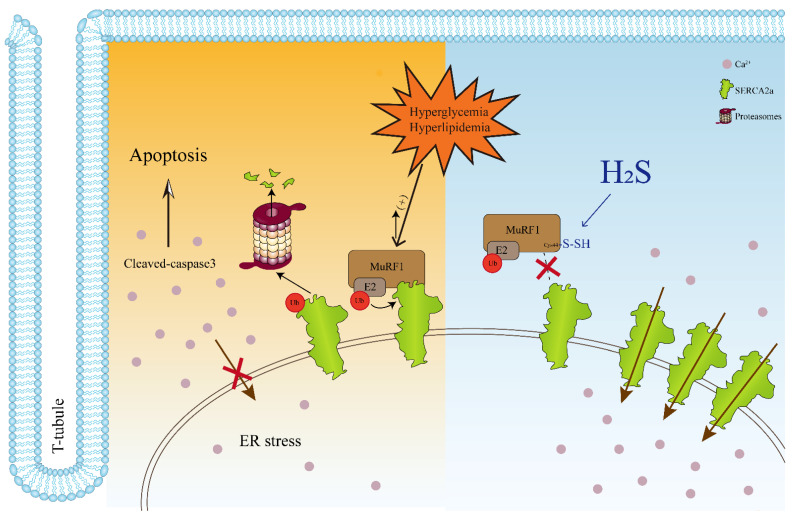
Model for the role of H_2_S in modifying MuRF1 S-sulfhydration to regulate SERCA2a ubiquitylation. In type 2 diabetic hearts, the endoplasmic reticulum protein SERCA2a protein is ubiquitinated by MuRF1, an E3 ubiquitin ligase, for proteasomal degradation. The increase of cytoplasmic calcium ion concentration leads to the decrease of myocardial contractile function, the decrease of endoplasmic reticulum calcium ion causes ER stress, and the excessive ER stress induces cell apoptosis. Meanwhile, H_2_S mediates S-sulfhydration at the Cys44 site of MuRF1, which reduces the ubiquitination level of SERCA2a, protects myocardial contractile function, and reduces the level of apoptosis.

This study aimed to elucidate the pathophysiological role of H_2_S and delineate the underlying mechanisms regulating cardiac contractile function and cardiomyocyte apoptosis in T2DM. Our data demonstrated that the H_2_S content and CSE expression were decreased in T2DM animal and cellular models. We also found that SERCA2a expression and activity were reduced; however, the ubiquitylation level of SERCA2a was increased in the cardiac tissues of the db/db mice, according to LC–MS/MS analysis. When treated with exogenous H_2_S, cardiac function was recovered, and cardiomyocyte apoptosis was decreased. Moreover, H_2_S reduced the ubiquitylation level of SERCA2a through MuRF1 S-sulfhydration to restore the protein levels and activity of SERCA2a. These findings allowed us to propose the following paradigm for the regulation of cardiac contractility and cardiomyocyte apoptosis in db/db mice treated with H_2_S: H_2_S → db/db mice → MuRFl S-sulfhydration ↑→ SERCA2a ubiquitylation ↑→ [Ca^2+^]_i_ ↑→ contractility ↑ and cardiomyocyte apoptosis↓, as schematically illustrated in Figure 8. Fractional calcium release, sarcoplasmic reticulum load, and cytosolic calcium sequestration play a crucial role in calcium homeostasis and calcium transients. Increasing evidence shows decreased contractile function with decreased SERCA2a protein levels [3]. The present study showed that the expression and activity of SERCA2a were decreased in the db/db mice, which resulted in a decrease in cardiac contractile function and an increase in cardiomyocyte apoptosis.

According to available SERCA2a crystal structures, the SERCA2a Ca^2+^ transport mechanism is well described at the functional and structural levels [30,31]. Three cytosolic domains include the nucleotide-binding domain, phosphorylation domain, and actuator domains (regulating ATP binding, autophosphorylation, and Ca^2+^ binding and translocation). SERCA2a is regulated by various posttranslational modifications (PTMs). In SERCA2a, ubiquitination sites (Lys 169, Lys 460, Lys 476, Lys 514, Lys 533, Lys 541, Lys 611, Lys 628, Lys 650, Lys 757, and Lys 995) have been reported. SERCA2a Lys460 is located at the nucleotide-binding domain, which regulates ATP binding. Using LC-MS/MS analysis, we identified 147 proteins that were hyperubiquitinated in the cardiac tissues of db/db mice compared to those treated with NaHS, including ATPase activity-related proteins in the sarcoplasmic reticulum membrane. SERCA2a ubiquitylation was obviously higher in the hearts of db/db mice than in those treated with NaHS.

In this study, MG132, a proteasome inhibitor, decreased the degradation of SERCA2a in the NRCMs treated with 40 mM glucose, 200 μM palmitate, and 200 μM oleate. We also constructed a point mutant plasmid of SERCA2a Lys460 to alanine, and the activity of SERCA2a was not different among the control, HG + pal + ole, and NaHS-treated groups after transfection of the SERCA2a-K460A overexpression plasmid. These results revealed that the ubiquitylation level of SERCA2a was increased in the hearts of the db/db mice, which led to a decrease in SERCA2a expression and activity.

MuRF1 is a muscle-specific E3 ubiquitin ligase that is identified as a RING finger protein. The structure of MuRF1 includes three domains: the RING, B-box, and coiled-coiled (CC) domains. The RING domain is responsible for binding to E2 enzymes and promoting ubiquitin transfer [32,33]. It can bind to a variety of substrates, including giant myofibrillar protein titin, myosin heavy chain (MyHc), and the autophagy-associated protein p62. In our results, we found that MuRFl could be combined with SERCA2a via immunoprecipitation to increase the ubiquitylation of SERCA2a.

H_2_S is a signaling molecule under physiological conditions. At pH 7.4, 80% of H_2_S is dissociated to the HS^−^ anion, and only 20% remains undissociated. Hydropersulfide (RSSH) is formed by the oxidation of thiols. In fact, the S-sulfhydration reaction can occur only when the cysteine group is in the oxidized form [34]. S-sulfhydration appears to be the principal posttranslational modification elicited by H_2_S. In our study, the H_2_S content, the protein level of CSE, and the level of hydropersulfide were decreased in the cardiac tissues of the db/db mice and the NRCMs treated with high glucose, palmitate, and oleate, whereas administration of exogenous H_2_S restored the H_2_S content, CSE expression, and the level of hydropersulfide. We also revealed that NaHS enhanced the S-sulfhydration of MuRFl, but DTT, a blocker of S-sulfhydration, reduced MuRFl S-sulfhydration. After MuRFl was mutated at Cys44, the level of S-sulfhydration in MuRFl was significantly decreased.

In conclusion, this study demonstrated that H_2_S enhanced MuRFl S-sulfhydration at cysteine 44 to modulate the ubiquitylation level of SERCA2a to restore the activity of SERCA2a in the hearts of the db/db mice. H_2_S may be a useful therapeutic strategy in DCM.

## Data Availability

The data presented in this study are available on request from the corresponding authors.

## Supplementary Materials

The following supporting information can be downloaded at: https://www.mdpi.com/article/10.3390/cells11213465/s1, Figure S1: Characterization of the experimental animal models; Figure S2: Exogenous H_2_S ameliorates endoplasmic reticulum stress in db/db mice.
