# Supplementary material for: Hydrogen Sulfide Regulates SERCA2a Ubiquitylation via Muscle RING Finger-1 S-Sulfhydration to Affect Cardiac Contractility in db/db Mice
Source: Cells. 2022 Nov 2;11(21):3465. doi: 10.3390/cells11213465 (PMC9658184; doi:10.3390/cells11213465)
Supplement: Supplementary file 1 [file cells-11-03465-s001.zip › cells-1958271-supplementary.pdf]

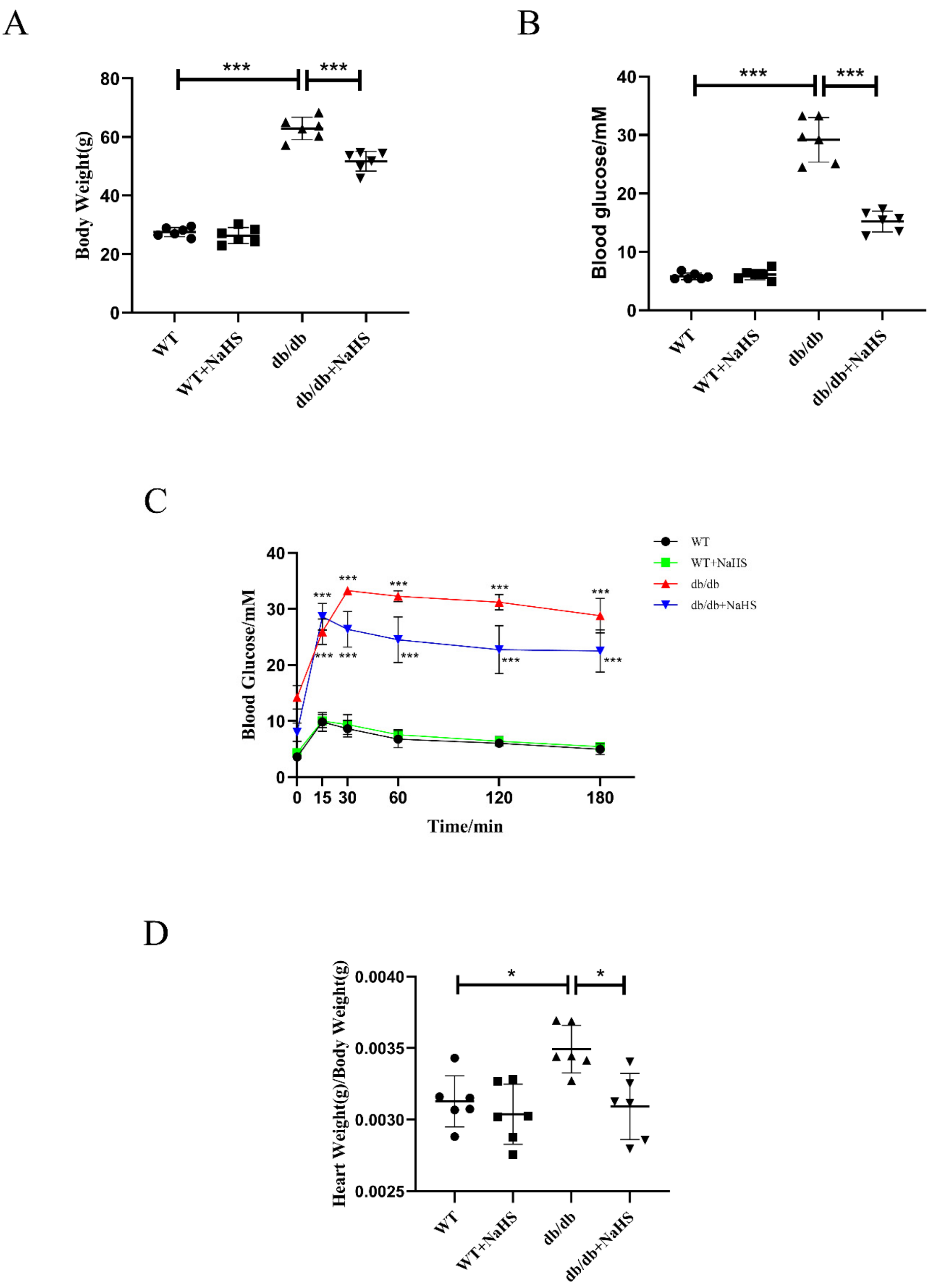

Figure S1. Characterization of the experimental animal models.

(A) The body weight of db/db mice in 26-week-treated db/db mice (*n*=6). **(B)** The blood glucose concentration of db/db mice in 26-week-treated db/db mice (*n*=6). **(C)** Intraperitoneal glucose tolerance test on four groups of mice, and injected with 2 g glucose/kg(*n*=4). **(D)** The ratio of heart weight/body weight in db/db mice (*n*=6). * $p < 0.05$, *** $p < 0.001$.

A

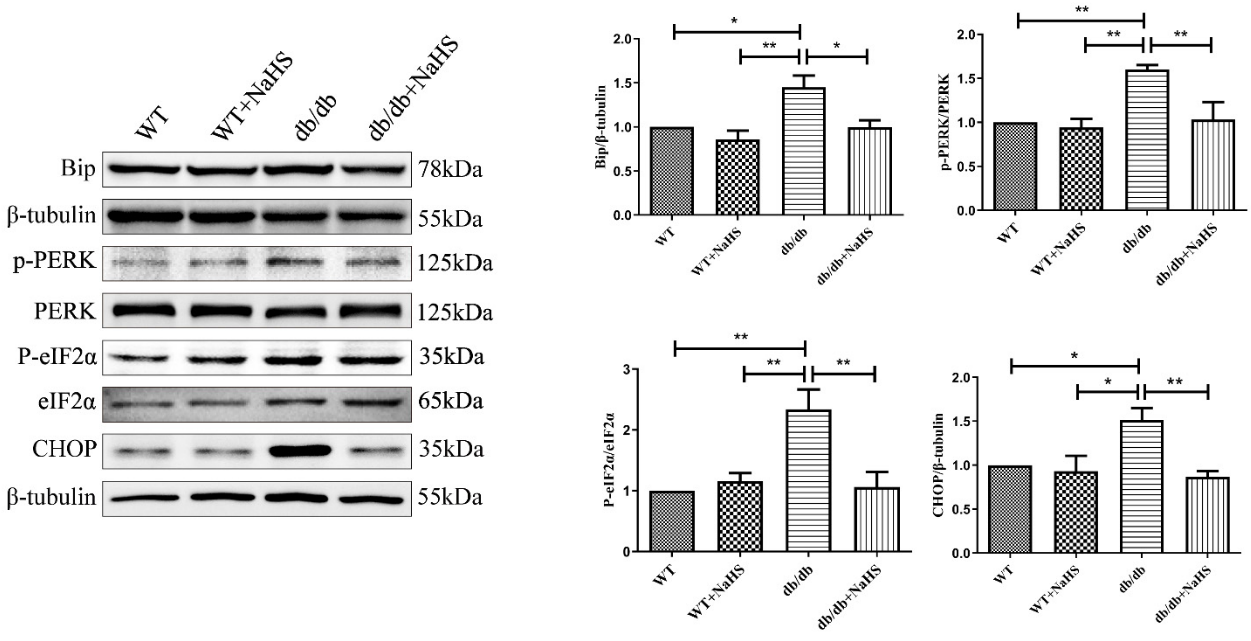

B

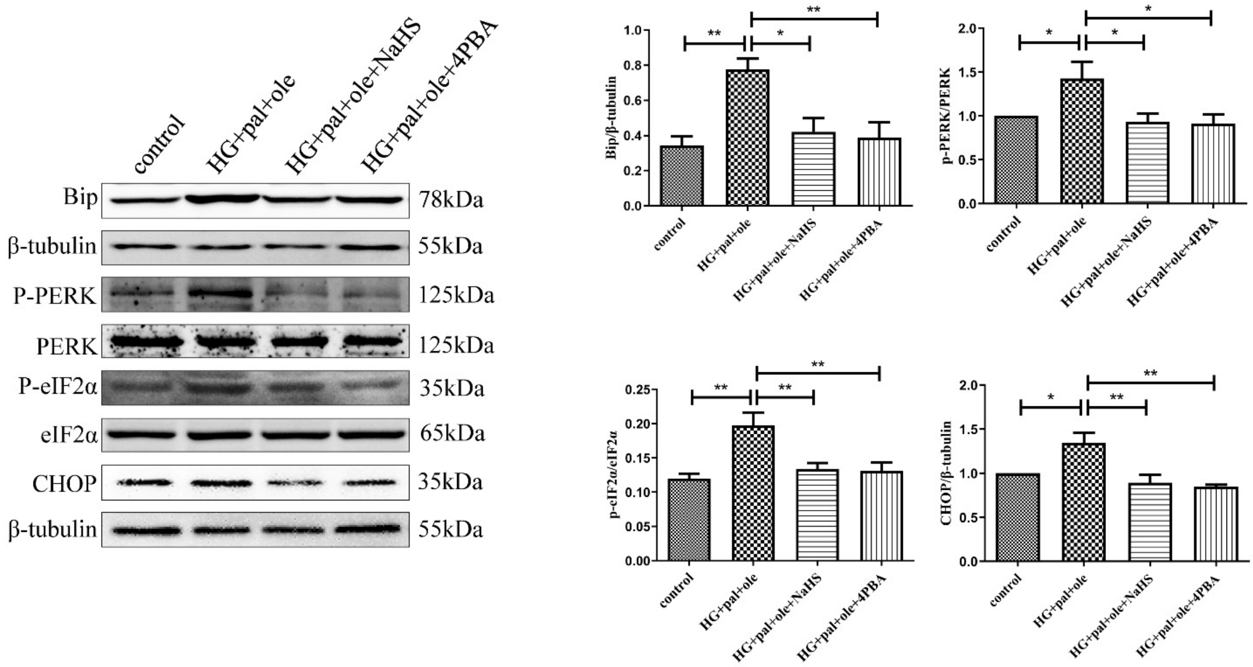

Figure S2. Exogenous H_2S ameliorates endoplasmic reticulum stress in db/db mice

(A) The expression level of ER stress associated protein was detected by Western blotting in db/db mice. **(B)** The expression level of ER stress associated protein was detected by Western blotting in NRCMs.

* $p < 0.05$, ** $p < 0.01$.
